# Using Wearable Devices to Examine the Associations of Sedentary Behavior with Perceived and Performance Fatigability Among Older Adults: The Study of Muscle, Mobility and Aging (SOMMA)

**DOI:** 10.3390/s25092722

**Published:** 2025-04-25

**Authors:** Reagan E. Garcia, Anne B. Newman, Eileen Johnson, Yujia Susanna Qiao, Peggy M. Cawthon, Barbara J. Nicklas, Bret H. Goodpaster, Nancy W. Glynn

**Affiliations:** 1 Department of Epidemiology, School of Public Health, University of Pittsburgh, Pittsburgh, PA 15261, USA; rem168@pitt.edu (R.E.G.); anewman@pitt.edu (A.B.N.); susannaqiao@pitt.edu (Y.S.Q.); 2 San Francisco Coordinating Center, California Pacific Medical Center Research Institute, San Francisco, CA 94107, USA; eileen.johnson@ucsf.edu (E.J.); peggy.cawthon@ucsf.edu (P.M.C.); 3 Department of Epidemiology and Biostatistics, University of California, San Francisco, CA 94158, USA; 4 Department of Internal Medicine, Section on Gerontology and Geriatric Medicine, Wake Forest University School of Medicine, Winston-Salem, NC 27157, USA; bnicklas@wakehealth.edu; 5 Translational Research Institute for Metabolism and Diabetes, Advent Health, Orlando, FL 32804, USA; bret.goodpaster@adventhealth.com

**Keywords:** aging, fatigue, inactivity

## Abstract

Fatigability, a phenotype of poor energy regulation, is associated with lower physical activity in older adults, but independent associations with sedentary behavior are unknown. We examined whether sedentary behavior was associated with fatigability using cross-sectional data from the Study of Muscle, Mobility and Aging. Mean sedentary time, sedentary bout length, and sedentary breaks/day were measured using 7-day waking hour data collected from a thigh-worn accelerometer. Fatigability was assessed using the Pittsburgh Fatigability Scale Physical subscale (PFS, score 0–50, higher = greater fatigability) and the Pittsburgh Performance Fatigability Index (PPFI), a percentage decline of observed cadence to maximal cadence from a wrist-worn accelerometer captured during a usual-paced 400 m walk (range 0–100%, higher = more performance deterioration). The participants (N = 663; 76.4 ± 5.1 years, 58% women, 54% high PFS, median PPFI 1.4%) were sedentary for 614.8 ± 111.7 min/day, with a mean 15.0 ± 5.5 min/day bout length and mean 46.1 ± 13.2 sedentary breaks/day. Higher total sedentary time was associated with greater PFS Physical score (β = 0.71, *p* = 0.0368), but this association was not independent of step count/day. After adjusting for step count/day, higher sedentary time was associated with lower PPFI score (β = −0.44, *p* = 0.0039). Sedentary bout length and breaks/day were not associated with perceived or performance fatigability. Future studies should aim to better understand the inter-relatedness of these behaviors.

## 1. Introduction

Fatigability, a trait that contextualizes whole-body fatigue to the level of activity (i.e., intensity and duration) with which the fatigue is associated, is an important prognostic factor associated with mobility decline, many chronic diseases, and mortality among older adults [1,2,3]. In particular, fatigability is implicated in the mobility disability pathway and may be a sign of accelerated aging [4]. Both perceived fatigability, one’s perception of their susceptibility to fatigue, and performance fatigability, one’s performance deterioration during a physical task, are prevalent among older adults, with prevalence rates as low as 20% in younger (60–70 years), healthier older adults and as high as 90% among older adults ≥90 years old [1]. While the biological pathways leading to fatigability are not yet fully understood, previous studies suggest that greater perceived and performance fatigability in older adults may rise from increasing energy deficits via lower skeletal muscle ATP production [5,6], lower physical fitness [7,8], and/or less physical activity [9,10,11,12,13]. In particular, lower levels of moderate–vigorous physical activity are associated with greater perceived and performance fatigability [9,10,12], and increasing time spent in moderate-vigorous activity can decrease perceived fatigability [14,15,16]. Higher activity fragmentation is also associated with greater perceived fatigability [17,18]. In response, lifestyle interventions, particularly those designed to increase physical activity and decrease sedentary time, have been proposed as a potential target to reduce fatigability and therefore extend the healthspan of the aging population [14].

Sedentary behavior, a physical behavior classified by low energy expenditure (≤1.5 Metabolic Equivalent Tasks, METs) in a reclining or sitting posture, is distinct from physical inactivity, defined as failing to meet the World Health Organization’s Physical Activity Guidelines of 150 min of moderate-vigorous aerobic activity per week [19]. Because sedentary behavior and inactivity are distinct, older adults can be both physically active and highly sedentary. For example, an older adult may walk at a moderate-intensity pace for 25 min each morning, meeting a weekly goal of 150 min of physical activity, but may spend the following several hours sitting reading, which is a highly sedentary activity. Because one can be both physically active and highly sedentary, understanding the associations between sedentary behavior and fatigability is crucial to understand whether low energy expenditure behaviors can independently influence one’s perceived or performance fatigability, regardless of their physical activity habits.

Qiao et al. assessed the prospective association between arm-worn monitor-measured sedentary behavior and perceived fatigability in older men and found no association [10]. However, this study was limited in that the wearable device used did not distinguish posture, a key component of sedentary behavior. Using a thigh-worn accelerometer is recommended to measure sedentary behavior as this placement site is able to more accurately distinguish postures than other placement sites, notably the waist and wrist [20,21,22]. Additionally, Qiao et al. did not assess how sedentary time was accumulated. Longer sedentary bouts or greater transitions between sitting, standing, and/or stepping may have different associations with fatigability. To our knowledge, no studies have examined these associations in older women, who report higher fatigability than men [1]. Additionally, no studies have evaluated the association between sedentary behavior and performance fatigability. Recently, the Pittsburgh Performance Fatigability Index, which uses acceleration data from a wrist-worn accelerometer to estimate the cadence-vs-time trajectory, was validated as a more sensitive measure of performance fatigability [23]. Using this novel wearable device measure of performance fatigability provides an ability to detect any association between sedentary behavior and small performance decrements.

Therefore, we examined the cross-sectional associations of sedentary behavior using measures from an activPAL accelerometer worn on the thigh with both perceived and performance fatigability in the Study of Muscle, Mobility and Aging (SOMMA). SOMMA is a unique prospective cohort which contains many novel measures among older adults. We examined multiple characterizations of sedentary behavior, including total sedentary time, mean sedentary bout length, and number of sit-to-stand transitions (herein called sedentary breaks/day) [24]. We hypothesized that greater total sedentary time and longer mean sedentary bout length would be associated with higher perceived and performance fatigability scores, while more sedentary breaks/day would be associated with lower perceived and performance fatigability scores.

## 2. Materials and Methods

### 2.1. Study Population

**Figure 1:**
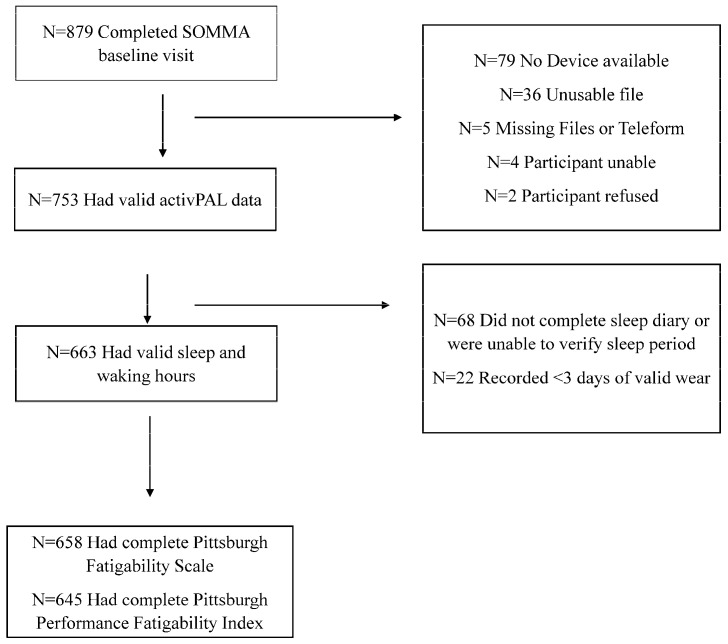
Flowchart of Inclusion into Secondary Analysis of Baseline Data: The Study of Muscle, Mobility and Aging (SOMMA).

Community-dwelling older adults (N = 879) were recruited for SOMMA from two locations (University of Pittsburgh and Wake Forest University School of Medicine) between April 2019 and December 2021. The prospective cohort study design has been described in detail elsewhere [25]. Briefly, participants were eligible to participate in the baseline SOMMA visit if they were aged 70 years or older with a body mass index (BMI) of <40 kg/m^2^ and agreed to undergo muscle tissue biopsy and magnetic resonance scans. Additionally, eligible participants had to be able to complete a usual-paced 400 m walk in <15 min. The participants who did not appear to be able to complete a 400 m walk were asked to complete a 4 m walk and were excluded if 4 m gait speed was <0.6 m/s. The exclusion criteria also included self-reported inability to walk one-quarter mile (i.e., 400 m) or climb a flight of stairs, active malignancies or dementia, or medical contraindication to biopsy or magnetic resonance scans. All participants gave informed written consent. The Western IRB-Copernicus Group Institutional Review Board (WCG-IRB, study number 20180764) approved the study as the central IRB. The participants completed a series of baseline examinations and questionnaires over three to four days with the goal of having all assessments collected within 6 weeks of each other. The final sample for this cross-sectional analysis included 663 participants who had valid activPAL data and scored sleep time (to identify out-of-bed intervals) at baseline (Figure 1).

### 2.2. Perceived and Performance Fatigability Measures

Perceived fatigability was measured using the validated Pittsburgh Fatigability Scale (PFS) [26,27]. The PFS is a self-administered 10-item scale that asks participants to rate the level of physical and mental fatigue they expect or imagine they will feel after performing each task (0 = no fatigue, 5 = extreme fatigue). Each of the 10 items implied a specific intensity and duration, and items ranged from low to high intensity (e.g., sitting quietly for one hour, brisk or fast walk for 1 h). Physical and Mental scores ranged from 0 to 50, with higher scores indicating greater perceived fatigability. Only the PFS Physical subscale (10-items, range 0–50) was used for this analysis. Scores were imputed for participants missing 3 or fewer items (n = 10) [28].

Performance fatigability was assessed using the Pittsburgh Performance Fatigability Index (PPFI) [23,29]. The participants wore an ActiGraph GT9X (Link) accelerometer (ActiGraph LLC, Pensacola, FL, USA) on their non-dominant wrist during a usual-paced 400 m Long Distance Corridor Walk. The participants were instructed to walk 10 laps on a 40 m course (20 m marked space with cones) at their normal pace. Raw accelerometer data collected at a frequency of 80 Hz were processed prior to the statistical analysis for this manuscript in R (version 4.0) to calculate PPFI [29]. PPFI was determined from the ratio comparing the area under an individuals observed cadence-versus-time trajectory during the 400 m walk to a hypothetical area that would be observed in the absence of fatigue [23]. Cadence trajectories were fitted for each participant using penalized regression splines. A higher PPFI score (range 0% to 100%) indicates more severe performance fatigability [23].

### 2.3. Sedentary Behavior Measurement

Sedentary behavior was measured using the activPAL4^TM^ micro (PAL Technologies Ltd., Glasgow, Scotland). The activPAL is a triaxial accelerometer with a built-in inclinometer. The accelerometer measures accelerations across three axes, while the inclinometer distinguishes activity spent sitting or reclining, standing, and stepping. At baseline (generally on the same day that the 400 m walk was completed), the activPAL was placed on the midline of the participant’s right thigh using 3 M Tegaderm. The participants were asked to wear the activPAL for 7 consecutive 24 h periods (Valid wear ≥3 days of 10+ hours of wear per day) and complete a sleep diary. Time in bed, determined by the self-reported sleep and wake times from the sleep diary, were excluded from the calculation of each sedentary behavior variable. The standard activPAL algorithm was used to classify the data into non-upright, standing, and stepping events using the inclinometer. For each valid day, total sedentary time (min/day), mean sedentary bout length (min/day), and number of sedentary breaks/day during waking hours were calculated and averaged over the entire wear period. We also generated quartiles of each sedentary variable to examine the strength of the association with fatigability across different doses of sedentary behavior given the physical activity literature, which shows a dose–response relationship between physical activity and fatigability [10,12]. Daily step count, derived using the dynamic acceleration of the thigh and foot strikes, was defined as mean number of steps per day. Of the 753 participants who wore the activPAL device at baseline, 68 did not complete sleep diaries and 22 did not meet valid wear time criteria, leading to a final sample size of 663 participants with valid activPAL data (Figure 1). There were no differences between those with valid activPAL data and those without.

### 2.4. Confounders

The following variables were considered as potential confounders because of their associations with sedentary behavior and all measures of fatigability, namely age, sex, multimorbidity, anthropometrics, peak oxygen consumption, and step count/day. Age at baseline, sex, and race/ethnicity (Non-Hispanic White vs. Racial/Ethnic Minority) were self-reported. Height (m) without shoes was measured via stadiometers and weight (kg) with light clothing was measured on digital scales which were then used to calculate body mass index (BMI, kg/m^2^). A composite multimorbidity index was created using a modified list of chronic conditions from the Rochester Epidemiology Project [30]. Items included history (yes/no) of self-reported, physician diagnosed conditions: cancer, chronic kidney disease or renal failure, atrial fibrillation, lung disease (i.e., COPD [chronic obstructive pulmonary disease], bronchitis, asthma, or emphysema), coronary heart disease (i.e., blocked artery or myocardial infarction), heart failure, dementia, diabetes, stroke, aortic stenosis, and depressive symptoms (measured using the CESD-10, range 0–30, a higher score indicates more depressive symptoms). Multimorbidity was categorized as 0 condition, 1 condition, or 2 or more conditions. The participants completed a three-stage cardiopulmonary exercise treadmill test to measure oxygen consumption (VO_2_, mL/ min) [31]. After walking for 5 min at their preferred pace (derived from 400 m walk speed), the participants completed a modified Balke or manual symptom-limited maximal test. VO_2_peak was expressed as absolute (mL/min) and relative to bodyweight (mL/kg/min). Step count/day was measured using average daily step count/day from the activPAL accelerometer.

### 2.5. Statistical Analyses

Descriptive characteristics were generated using mean ± standard deviation (SD) or frequencies for normally distributed variables. Normality was assessed visually using density and QQ plots. Variables with non-normal distributions were represented using median (interquartile range). Sedentary behavior variables were assessed as continuous variables and by quartiles. ANOVA was used to examine differences in PFS Physical score and PPFI score across quartiles of each sedentary behavior. The Jonckheere–Terpstra test was used to assess whether there was a non-linear trend across quartiles; if *p* < 0.05, we generated linear regression models examining the behavior as a continuous variable with the beta expressed per SD unit and by quartiles of sedentary behavior. After examining linear regression model residuals, the normality assumption for PPFI was not met. Since the scores were left-censored at 0 and right-skewed, we used Tobit regression to examine each sedentary behavior variable with PPFI. The models were adjusted for variables that are associated with both perceived and performance fatigability and include age, sex, clinic site, height, weight, absolute peak oxygen consumption, and multimorbidity. To examine associations of sedentary behavior and fatigability independent of physical activity, we also ran models additionally adjusted for mean step count/day. Statistical analyses were conducted using R version 4.4.0. The SOMMA data release from November 2023 was used for these analyses.

## 3. Results

A total of 663 older adults (76.4 ± 5.1 years, 58% women, 85% Non-Hispanic White, step count/day 6928 ± 3120) had valid activPAL data at baseline (Table 1). The participants spent on average 614.8 ± 111.7 min/day (10.2 ± 1.8 h/day) in sedentary time with mean sedentary bout length of 15.0 ± 5.5 min/day. The participants also had on average 46.1 ± 13.2 sedentary breaks/day (Table 1). Over half (54.0%) of the participants reported more severe perceived physical fatigability (PFS Physical score ≥ 15). The median PPFI score was 1.4% (Table 1). The descriptive characteristics by quartiles of mean sedentary bout length and sedentary breaks/day can be found in the Supplementary Materials (Tables S1 and S2).

Every 112 min/day more sedentary time was associated with a 0.71 ± 0.34 (*p* = 0.0368) higher PFS Physical score after adjustment (Table 2). Continuous associations of mean sedentary bout length and sedentary breaks/day with PFS Physical score were not statistically significant after adjustment (Table 2). Additionally, adjusting for step count/day attenuated all associations between sedentary time and PFS Physical score (*p* > 0.05) (Table S3). Sedentary time, sedentary bout length, and sedentary breaks/day were not associated with continuous PPFI score in initial models (Table 3). However, after adjusting for step count, one standard deviation higher sedentary time was associated with lower performance fatigability (PPFI β =−0.44 ± 0.15, *p* = 0.0039) (Table S4).

**Figure 2:**
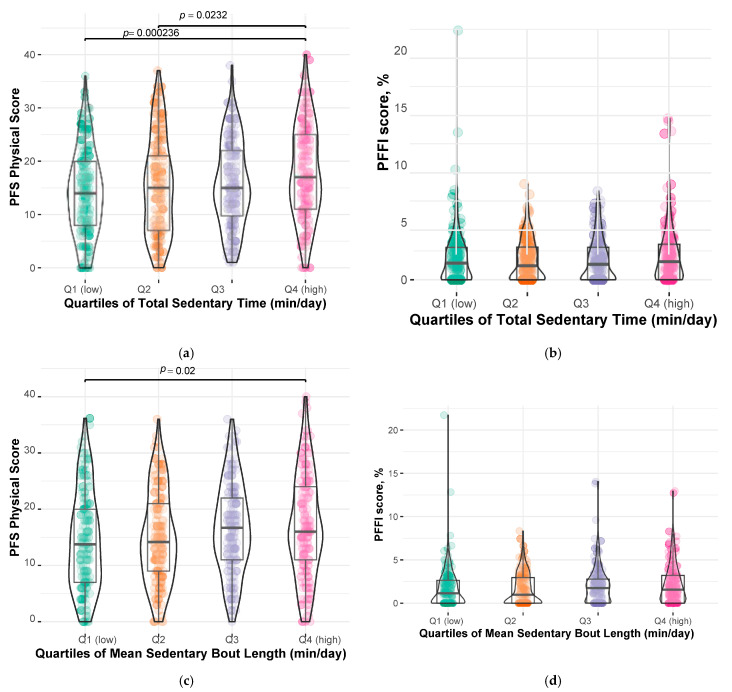

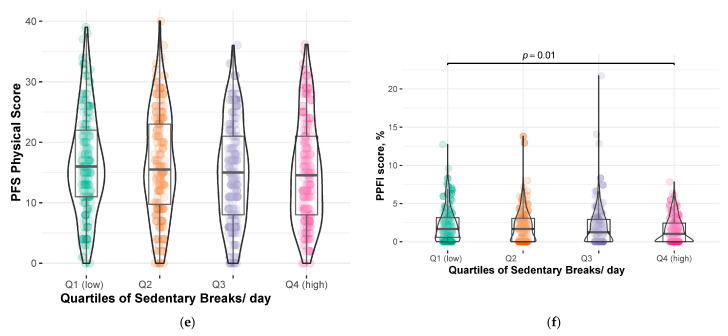
Perceived and Performance Fatigability scores by Quartiles of Explanatory Sedentary Variables: The Study of Muscle, Mobility and Aging (SOMMA). (**a**) difference in Pittsburgh Fatigability Scale (PFS) Physical score by quartiles of total sedentary time; (**b**) difference in Pittsburgh Performance Fatigability Index (PPFI) score by quartiles of total sedentary time; (**c**) difference in PFS Physical score by quartiles of mean sedentary bout length; (**d**) difference in PPFI score by quartiles of mean sedentary bout length; (**e**) difference in PFS Physical score by quartiles of sedentary breaks/day; and (**f**) difference in PPFI score by quartiles of sedentary breaks/day.

Overall ANOVA revealed differences in PFS Physical score by quartiles of total sedentary time and mean sedentary bout length, but not sedentary breaks/day (Figure 2). No differences in PPFI score were observed across quartiles of total sedentary time and mean sedentary bout length. However, the PPFI scores were statistically different across quartiles of sedentary breaks/day (Figure 2). The Johnckheere–Terpstra test indicated potential non-linearities for total sedentary time and mean sedentary bout length (*p* < 0.001) but was not significant for sedentary breaks/day (*p* > 0.05) for both PFS Physical and PPFI scores. For this reason, we did not build regression models using sedentary breaks/day as quartiles with PFS Physical score or PPFI score.

When examining sedentary time as quartiles, participants with the greater (Q4) total sedentary time had on average 2.67 ± 0.94 (*p* = 0.0044) higher PFS Physical score compared to the participants with the least sedentary time (Q1) after covariate adjustment (Table 2). PFS Physical score did not significantly vary across quartiles of mean sedentary bout length (min/day) (Table 2). The participants in Q3 had lower PPFI scores (i.e., less performance deterioration) compared to the participants with the least sedentary time (Q1) (−1.12 ± 0.39, *p* = 0.0039, Table 3). When additionally adjusting for step count/day, participants with higher sedentary time (Q3 and Q4) had significantly lower PPFI scores compared to the participants with the least sedentary time (Q1), *p* < 0.05 (Table S4). No associations between sedentary bout length and PPFI score were observed (Table 3 and Table S4).

## 4. Discussion

In a cohort of community-dwelling older adults, total sedentary time, bout length, and sedentary breaks/day were largely unrelated to perceived fatigability particularly after adjustment for physical activity. Associations of sedentary time, bout length, and sedentary breaks/day had a nuanced association with performance fatigability. Our work highlights that sedentary behavior was not strongly associated with perceived or performance fatigability and may not be the optimal target by itself for future lifestyle intervention to reduce perceived or performance fatigability.

Our finding that sedentary time was not independently associated with perceived fatigability after accounting for physical activity is consistent with prior work reporting no association between sedentary behavior and perceived fatigability among older men, after accounting for total time spent in physical activity (MET ≥ 1.5) [10]. Both physical fatigability and sedentary behavior may be consequences of poor energy regulation [1,2,7,32], but they are influenced by levels of physical activity, a downstream effect of skeletal muscle energetics [9,10,13,14,23,33]. Sedentary behavior could also result from external factors, such as sedentary hobbies or lack of motivation to move. Since this is a cross-sectional analysis, temporality cannot be established. As such, it is plausible that more time spent sedentary may contribute to greater perceived physical fatigability or vice versa. Older adults may not be utilizing their energy reserve while sedentary and thus may perceive higher levels of fatigue. However, it is also possible that greater perceived physical fatigability may be hindering one’s motivation or perception of their abilities to perform more light or moderate-vigorous physical activities and may lead to more time spent in sedentary activities. When accounting for step count/day, associations between sedentary time and PFS Physical score became non-significant, suggesting that the association of sedentary behavior with perceived physical fatigability is not independent of total physical activity. However, physical behaviors are highly interrelated as higher sedentary time may come at the cost of lower moderate-vigorous physical activity, light physical activity, or sleep during a 24 h period. Future work is warranted to understand the benefits of substituting sedentary behavior with other active behaviors on reducing perceived physical fatigability.

Interestingly, total sedentary time and performance fatigability were not associated until step count/day were added to the continuous measure regression models. Unlike perceived fatigability, which is a perception of one’s capacity (i.e., what one thinks they can do), performance fatigability (i.e., what one does do) directly measures physical responses to fatigue, e.g., slowing down during a walking task, and may be more influenced by greater energy reserves. As sedentary behavior is only modestly associated with both cardiovascular fitness and measures of physical performance [34,35,36,37], it is unsurprising that total sedentary time was not associated with performance fatigability before adjustment for total physical activity. SOMMA is a highly active older adult cohort, as even those in the highest quartile of total sedentary time accumulated a mean 5124 steps/day. Normative data from the National Health and Nutrition Examination Survey suggests that the average steps/day for US adults 60 years or older range from 2749 to 4490 steps/day, demonstrating the high activity levels of SOMMA [38]. It is possible that older adults who are both active and highly sedentary may have had a greater energy reserve to perform the walking task without any performance decrement. Research between the associations of sedentary time and step count is limited yet suggests that these measures are somewhat associated [33]. To our knowledge, our study is the first to assess these behaviors jointly in relation to any measure of performance fatigability. Our nuanced findings of greater sedentary time being associated with lower performance fatigability indicate more research is needed to better elucidate the associations of sedentary time, step count, and performance fatigability, particularly among a less active cohort of older adults.

We did not find any significant associations between sedentary bout length or sedentary breaks/day with perceived or performance fatigability after adjustment for potential confounders. Our results suggest that overall sedentary time, but not sedentary patterns, may contribute to greater perceived and performance fatigability and is consistent with prior literature showing weak or null associations with sedentary patterns and other health outcomes such as cardiometabolic health [23,39,40]. The metabolic costs of sedentary breaks/day, i.e., sit-to-stand transitions, are higher than sitting, and consecutive transitions require greater energy expenditure [41]. The energy needed to complete sedentary breaks/day likely comes from the anaerobic phosphocreatine system as the movement, particularly among older adults, requires a quick burst of energy lasting less than 10 s. More or less sedentary breaks/day, therefore, likely do not significantly impact overall energy use or disuse and may not represent impairments in energy production associated with other measures of physical behaviors and fatigability (e.g., impairments in the electron transport chain during aerobic energy production). However, previous studies have reported associations with activity fragmentation and perceived physical fatigability, which is inconsistent with our findings of a null association [17,18]. It is possible that equivocal findings may be in part due to the varying definitions of activity fragmentation. Sedentary breaks/day may be capturing postural changes with small bursts of energy use rather than the likelihood of going from a more active to a less active state, requiring large, chronic changes in energy production and use over time, and this may explain inconsistent findings with the previous literature.

Perceived physical fatigability is a sensitive, prognostic indicator of future disability among older adults. Therefore, it is important to identify strategies that may be used to improve perceived physical fatigability among older adults. We postulate that improving moderate-vigorous physical activity may be the best intervention target as previous work has shown strong associations between moderate-vigorous physical activity and perceived physical fatigability [10,12,13]. Additionally, limited intervention work shows that improving moderate-vigorous physical activity can improve perceived physical fatigability [14]. Physical behaviors, while distinct, are highly interrelated, making it difficult to assess the independent associations of each other. For example, more time spent in sedentary behavior may come at the cost of less time spent in moderate-vigorous physical activity. Therefore, despite the null results of our analysis when adjusting for step count, an understanding of the role of sedentary behavior within the context of a 24 h day is needed. Substituting sedentary time with light or moderate physical activity may lead to lower perceived and performance fatigability, possibly through greater improvement in fitness and/or function. Future work in SOMMA will use compositional analysis to better examine the effects of the 24 h activity composition on perceived and performance fatigability and will further elucidate whether sedentary behavior can be targeted to reduce perceived physical fatigability.

Our work has some limitations. First, our results may not generalize to ethnic/racial minority communities, as SOMMA participants were predominantly White non-Hispanic (85.1%). Next, residual confounding may still be present after adjustment for potential confounders. Our results are cross-sectional and observational which limits our ability to draw conclusions about causality and the directionality of our results. Our data also support the hypothesis that greater fatigability leads to increased sedentariness. Despite these limitations, strengths of this study included that sedentary behavior were measured using the activPAL accelerometer. The activPAL inclinometer accurately captures changes in posture and separates activities by reclining or sitting, standing, and stepping behaviors [42]. Additionally, both perceived and performance fatigability were measured using sensitive, validated methods [23,26,27,29].

Overall, while greater sedentary time was associated with greater perceived physical fatigability, no other metrics of sedentariness were, suggesting that some types of sedentary behavior may contribute to greater perceptions of fatigue. However, these associations were not independent of physical activity. Associations of sedentariness with performance fatigability were nuanced, suggesting the need to examine sedentary time in the context of the 24 h day. Future work should disentangle the temporality of the association between sedentariness and fatigability to inform the development of interventions to improve these outcomes important to older adults.

## Figures and Tables

**Table 1:** Baseline Descriptive Characteristics by Quartiles of Total Sedentary Time (min/day): The Study of Muscle, Mobility and Aging (SOMMA).

Characteristic	Total N = 663	Q1 (Low) [241.2, 547.6] n = 166	Q2 (547.6, 617.1] n = 166	Q3 (617.1, 691.6] n = 165	Q4 (High) (691.6, 897.7] n = 166	*p*-Trend
Age, years	76.4 ± 5.1	76.2 ± 5.1	76.3 ± 4.7	76.7 ± 5.4	76.6 ± 5.0	0.8
Sex, Women	387 (58.4)	118 (71.1)	99 (59.6)	97 (58.8)	73 (44.0)	<0.001
Race, Non-Hispanic White	563 (84.9)	141 (84.9)	147 (88.6)	137 (83.0)	138 (83.1)	0.5
Multimorbidity Index ^†^						0.2
No conditions	280 (42.8)	67 (40.9)	84 (50.9)	70 (42.9)	59 (36.4)	
One condition	262 (40.1)	71 (43.3)	60 (36.4)	62 (38.0)	69 (42.6)	
Two or more conditions	112 (17.1)	26 (15.9)	21 (12.7)	31 (19.0)	34 (21.0)	
Body Mass Index, kg/m^2^	27.7 ± 4.6	26.3 ± 4.8	27.1 ± 4.3	28.6 ± 4.8	28.7 ± 4.1	<0.001
Height, m	1.7 ± 0.1	1.6 ± 0.1	1.7 ± 0.1	1.7 ± 0.1	1.7 ± 0.1	<0.001
Weight, kg	76.3 ± 15.5	69.2 ± 14.1	74.7 ± 15.1	79.1 ± 15.0	82.1 ± 14.7	<0.001
VO_2_peak, mL/kg/min	20.0 ± 4.6	20.7 ± 4.7	20.8 ± 4.6	19.5 ± 4.8	19.0 ± 3.9	<0.001
VO_2_peak, mL/min	1517.6 ± 426.0	1426.0 ± 400.7	1561.0 ± 447.2	1531.7 ± 445.5	1552.0 ± 397.3	<0.001
Average 400 m Walk Speed, m/s	1.05 ± 0.18	1.06 ± 0.16	1.06 ± 0.17	1.05 ± 0.18	1.02 ± 0.19	0.2
Daily Step Count, /day	6928.3 ± 3120.0	8631.7 ± 3449.4	7483.1 ± 2951.6	6471.7 ± 2805.2	5123.9 ± 1977.3	<0.001
Total Sedentary Time, min/day	614.8 ± 111.7	467.6 ± 64.6	585.5 ± 19.5	655.8 ± 22.4	750.7 ± 45.7	<0.001
Total Standing Time, min/day	244.5 ± 90.7	350.2 ± 84.8	252.0 ± 54.0	203.6 ± 46.4	172.1 ± 50.2	<0.001
Sedentary Bout Length, min/day	15.0 ± 5.5	11.2 ± 3.2	13.6 ± 3.9	16.1 ± 4.7	19.1 ± 6.2	<0.001
Sedentary Breaks, /day	46.1 ± 13.2	46.0 ± 13.3	47.8 ± 13.2	46.3 ± 13.6	44.3 ± 12.6	0.094
PFS Physical score, 0–50	15.8 ± 8.5	14.1 ± 8.0	15.3 ± 9.0	15.7 ± 7.9	17.9 ± 8.8	0.001
PPFI score *, %	1.4 (0.0, 2.9)	1.4 (0.0, 2.8)	1.2 (0.0, 2.8)	1.3 (0.0, 2.9)	1.6 (0.0, 3.1)	0.5

^†^ The Multimorbidity Index considered 11 self-reported, physician diagnosed conditions: cancer, chronic kidney disease or renal failure, atrial fibrillation, lung disease, coronary heart disease, heart failure, dementia, diabetes, stroke, aortic stenosis, and depressive symptoms. * Median (IQR) for non-normally distributed variables. Note: VO_2_peak = Peak oxygen consumption; PFS = Pittsburgh Fatigability Scale; PPFI = Pittsburgh Performance Fatigability Index.

**Table 2:** Associations of Sedentary Behavior with Perceived Physical Fatigability: The Study of Muscle, Mobility and Aging (SOMMA, N = 658).

	1 Standard Deviation	β (Standard Deviation)	95% CI
Total Sedentary Time, min/day			
Continuous (per 1 standard deviation) ^†^	112	0.71 (0.34) *	0.04, 1.38
Q1 (lowest) **^#^**		Ref.	
Q2		1.47 (0.88)	−0.26, 3.20
Q3		0.75 (0.90)	−1.01, 2.51
Q4 (highest)		2.67 (0.94) *	0.84, 4. 51
Mean Sedentary Bout Length, min/day			
Continuous (per 1 standard deviation) ^†^	5.5	0.16 (0.33)	−0.49, 0.81
Q1 (lowest) **^#^**		Ref.	
Q2		0.49 (0.88)	−1.25, 2.22
Q3		1.19 (0.91)	−0.59, 2.97
Q4 (highest)		0.98 (0.94)	−0.87, 2.82
**Sedentary Breaks, /day**			
Continuous (per 1 standard deviation) ^†^	13.2	0.02 (0.32)	−0.60, 0.64

^†^ Separate models were generated using continuous and quartile sedentary behavior variables. * *p* < 0.05. ^#^ Quartiles are compared to Q1 (Quartile 1). Quartile ranges for total sedentary time (all min/day): Q1: 241.2467 to 547.6; Q2: >547.6 to 617.1081; Q3: >617.1081 to 691.5590; Q4: >691.5590 to 897.6678. Quartile ranges for mean sedentary bout length (all min/day): Q1: 4.2183 to 11.2369; Q2: >11.2369 to 14.1469; Q3: >14.1469 to 17.7291; Q4: >17.7291 to 51.7430. Notes: Linear regression models were adjusted for age, sex, clinic site, height (m), weight (kg), multimorbidity index, and absolute peak oxygen consumption (mL/min). Perceived fatigability was measured using the Pittsburgh Fatigability Scale Physical score.

**Table 3:** Associations of Sedentary Behavior with Performance Fatigability: The Study of Muscle, Mobility and Aging (SOMMA, N = 645).

	1 Standard Deviation	β (Standard Deviation)	95% CI
Total Sedentary Time, min/day			
Continuous (per 1 standard deviation) ^†^	111.7	−0.25 (0.14)	−0.54, 0.03
Q1 (lowest) **^#^**		Ref.	
Q2		−0.49 (0.38)	−1.23, 0.25
Q3		−1.12 (0.39) *	−1.88, −0.36
Q4 (highest)		−0.56 (0.40)	−1.34, 0.21
Mean Sedentary Bout Length, min/day			
Continuous (per 1 standard deviation) ^†^	5.4	−0.02 (0.14)	−0.29, 0.25
Q1 (lowest) **^#^**		Ref.	
Q2		−0.54 (0.39)	−1.29, 0.22
Q3		−0.16 (0.39)	−0.92, 0.60
Q4 (highest)		−0.28 (0.40)	−1.06, 0.50
Sedentary Breaks, /day			
Continuous (per 1 standard deviation) ^†^	13.2	−0.16 (0.14)	−0.43, 0.10

^†^ Separate models were generated using continuous and quartile sedentary behavior variables. * *p* < 0.05. ^#^ Quartiles are compared to Q1 (Quartile 1). Quartile ranges for total sedentary time (all min/day): Q1: 241.2467 to 547.6; Q2: >547.6 to 617.1081; Q3: >617.1081 to 691.5590; Q4: >691.5590 to 897.6678. Quartile ranges for mean sedentary bout length (all min/day): Q1: 4.2183 to 11.2369; Q2: >11.2369 to 14.1469; Q3: >14.1469 to 17.7291; Q4: >17.7291 to 51.7430. Notes: Tobit regression models were adjusted for age, sex, clinic site, height (m), weight (kg), multimorbidity index, and absolute peak oxygen consumption (mL/min). Performance Fatigability was measured using the Pittsburgh Performance Fatigability Index (PPFI).

## Data Availability

The original data presented in the study are openly available at https://sommaonline.ucsf.edu/. This manuscript used data that were part of the November 2023 release, accessed on 20 January 2024.

## Supplementary Materials

The following supporting information can be downloaded at https://www.mdpi.com/article/10.3390/s25092722/s1. Table S1: Descriptive characteristics by quartiles of mean sedentary bout length (min/day): The Study of Muscle, Mobility and Aging (SOMMA). Table S2: Descriptive characteristics by quartiles of sedentary breaks/day: The Study of Muscle, Mobility and Aging (SOMMA). Table S3: Associations of sedentary behavior with perceived physical fatigability adjusted for step count/day: the Study of Muscle, Mobility and Aging (SOMMA, N = 658). Table S4: Associations of sedentary behavior with performance fatigability, adjusted for step count/day: The Study of Muscle, Mobility and Aging (SOMMA, N = 645).

## Abbreviations

The following abbreviations are used in this manuscript:

SOMMA	Study of Muscle, Mobility and Aging
PFS	Pittsburgh Fatigability Scale
PPFI	Pittsburgh Performance Fatigability Index
