# Supplementary material for: Using Wearable Devices to Examine the Associations of Sedentary Behavior with Perceived and Performance Fatigability Among Older Adults: The Study of Muscle, Mobility and Aging (SOMMA)
Source: Sensors (Basel). 2025 Apr 25;25(9):2722. doi: 10.3390/s25092722 (PMC12074308; doi:10.3390/s25092722)
Supplement: Supplementary file 1 [file sensors-25-02722-s001.zip › sensors-3543204-supplementary.pdf]

Supplemental Material for:

Using Wearable Devices to Examine the Associations of Sedentary Behavior with Perceived and Performance Fatigability among Older Adults: The Study of Muscle, Mobility and Aging (SOMMA)

AUTHORS AND AFFILIATIONS

Reagan E. Garcia MPH[1], Anne B. Newman MD[1], Eileen Johnson MPH[2], Yujia Susanna Qiao PhD[1], Peggy M. Cawthon PhD[2,3], Barbara J. Nicklas PhD[4], Bret H. Goodpaster PhD[5], and Nancy W. Glynn PhD[1]

[1]Department of Epidemiology, School of Public Health, University of Pittsburgh, Pittsburgh, PA, USA.

[2]San Francisco Coordinating Center, California Pacific Medical Center Research Institute, San Francisco, CA, USA.

[3]Department of Epidemiology and Biostatistics, University of California, San Francisco, CA, USA.

[4]Department of Internal Medicine, Section on Gerontology and Geriatric Medicine, Wake Forest University School of Medicine, Winston-Salem, NC, USA.

[5]Translational Research Institute for Metabolism and Diabetes, Advent Health, Orlando, FL, USA.

Corresponding Author:
Nancy W. Glynn, PhD, FGSA, FACSM
epidnwg@pitt.edu
University of Pittsburgh School of Public Health
Department of Epidemiology, Center for Aging and Population Health
130 DeSoto Street, 5120 Public Health, Pittsburgh PA 15261

Table S1. Descriptive Characteristics by Quartiles of Mean Sedentary Bout Length (min/day): The Study of Muscle, Mobility and Aging (SOMMA)

Characteristic	Total N=663	Q1 (low) [4.2, 11.2] n=166	Q2 (11.2, 14.1] n=166	Q3 (14.1, 17.7] n=165	Q4 (high) (17.7, 51.7] n=166	P-trend
Age, years	76.4±5.1	76.0±4.9	76.3±5.0	76.5±5.3	76.9±5.0	0.3
Sex, Women	387 (58.4)	115 (69.3)	105 (63.3)	88 (53.3)	79 (47.6)	<0.001
Race, Non-Hispanic White	563 (84.9)	138 (83.1)	144 (86.7)	142 (86.1)	139 (83.7)	0.8
Multimorbidity Index†						0.4
No conditions	280 (42.8)	71 (43.8)	81 (49.1)	64 (39.5)	64 (38.8)	
One condition	262 (40.1)	67 (41.4)	61 (37.0)	67 (41.4)	67 (40.6)	
Two or more conditions	112 (17.1)	24 (14.8)	23 (13.9)	31 (19.1)	34 (20.6)	
Body Mass Index, kg/m^2	27.7±4.6	25.6±4.4	27.6±4.5	28.2±4.5	29.3±4.3	<0.001
Height, m	1.7±0.1	1.6±0.1	1.7±0.1	1.7±0.1	1.7±0.1	0.001
Weight, kg	76.3±15.5	68.7±14.2	75.6±14.8	78.4±15.1	82.5±14.4	<0.001
VO_2peak, mL/kg/min	20.0±4.6	21.1±4.7	20.6±4.5	19.5±4.5	18.7±4.2	<0.001
VO_2peak, mL/min	1,517.6±426.0	1,445.5±407.9	1,560.2±444.3	1,525.5±440.1	1,540.6±405.2	<0.001
Average 400m Walk Speed, m/s	1.05±0.18	1.07±0.16	1.07±0.18	1.04±0.17	1.01±0.18	0.001
Mean Total Step Count, /day	6,928.3±3,120.0	8,836.6±3372.7	7,413.2±2.787.0	6,404.1±2646.8	5,056.1±2290.9	<0.001
Total Sedentary Time, min/day	614.8±111.7	527.4±102.2	590.2±92.1	644.4±91.7	697.5±81.9	<0.001
Total Standing Time, min /day	244.5±90.7	301.6±98.0	256.7±85.2	231.1±76.6	188.5±59.7	<0.001
Mean Sedentary Bout Length, min/day	15.0±5.5	9.3±1.5	12.6±0.9	15.7±1.0	22.4±4.7	<0.001
Sedentary Breaks, /day	46.1±13.2	59.5±13.8	48.8±8.3	42.4±6.8	33.7±6.2	<0.001
PFS Physical score, 0-50	15.8±8.5	14.5±8.7	14.9±8.1	16.6±8.0	17.0±9.1	0.018
PPFI*, %	1.4 (0.0, 2.9)	1.1 (0.0, 2.7)	1.0 (0.0, 3.0)	1.7 (0.0, 2.7)	1.6 (0.0, 3.2)	0.05

*Median (IQR) for non-normally distributed variables

†Multimorbidity Index considered 11 self-reported, physician diagnosed conditions: cancer, chronic kidney disease or renal failure, atrial fibrillation, lung disease, coronary heart disease, heart failure, dementia, diabetes, stroke, aortic stenosis, and depression symptoms

VO_2peak = peak oxygen consumption; PFS = Pittsburgh Fatigability Scale; PPFI = Pittsburgh Performance Fatigability Scale

Table S2. Descriptive Characteristics by Quartiles of Sedentary Breaks/day: The Study of Muscle, Mobility and Aging (SOMMA)

**Characteristic**	**Total** N=663	**Q1 (low)** [14.4, 37.1] **n=167**	**Q2** (37.1, 44.5] **n=166**	**Q3** (44.5,53.1] **n=165**	**Q4 (high)** (53.1,110.1] **n=165**	**P-trend**
Age, years	76.4±5.1	76.9±5.0	76.7±5.3	75.7±4.6	76.4±5.3	0.14
Sex, Women	387 (58.4)	85 (50.9)	105 (63.3)	92 (55.8)	105 (63.6)	0.05
Race, Non-Hispanic White	563 (84.9)	140 (83.8)	143 (86.1)	144 (87.3)	136 (82.4)	0.6
Multimorbidity Index†						0.023
No conditions	280 (42.8)	58 (34.9)	73 (44.2)	85 (51.8)	64 (40.3)	
One condition	262 (40.1)	69 (41.6)	71 (43.0)	55 (33.5)	67 (42.1)	
Two or more conditions	112 (17.1)	39 (23.5)	21 (12.7)	24 (14.6)	28 (17.6)	
Body Mass Index, kg/m^2	27.7±4.6	28.7±4.6	28.1±4.8	27.5±4.4	26.3±4.4	<0.001
Height, m	1.7±0.1	1.7±0.1	1.7±0.1	1.7±0.1	1.6±0.1	0.2
Weight, kg	76.3±15.5	79.3±15.3	76.6±14.8	77.1±15.8	72.0±15.1	<0.001
VO_2peak, mL/kg/min	20.0±4.6	18.9±4.2	19.7±4.4	20.5±4.9	20.9±4.5	<0.001
VO_2peak, mL/min	1,517.6±426.0	1,498.5±406.7	1,501.2±408.3	1,572.5±465.2	1,497.6±420.0	<0.001
Average 400m Walk Speed, m/s	1.05±0.18	1.01±0.17	1.05±0.17	1.06±0.18	1.07±0.18	0.002
Mean Total Step Count, /day	6,928.3±3,120.0	5,467.7±2474.0	6,749.5±2787.8	7,424.9±3290.3	8,089.9±3262.2	<0.001
Total Sedentary Time, min/day	614.8±111.7	624.2±121.5	610.9±118.3	620.5±102.9	603.6±102.4	0.12
Total Standing Time, min /day	244.5±90.7	237.7±102.7	251.5±94.6	237.4±83.6	251.5±79.6	0.029
Mean Sedentary Bout Length, min/day	15.0±5.5	21.2±5.8	15.5±3.2	13.2±2.3	10.1±2.2	<0.001
Sedentary Breaks, /day	46.1±13.2	31.2±4.4	41.1±2.0	48.8±2.5	63.5±10.5	<0.001
PFS Physical Score, 0-50	15.8±8.5	16.6±8.5	16.3±8.7	15.0±8.4	15.1±8.5	0.2
PPFI*, %	1.4 (0.0, 2.9)	1.7 (0.6, 3.2)	1.7 (0.0, 3.1)	1.2 (0.0, 2.9)	1.0 (0.0, 2.4)	0.01

*Median (IQR) for non-normally distributed variables

†Multimorbidity Index considered 11 self-reported, physician diagnosed conditions: cancer, chronic kidney disease or renal failure, atrial fibrillation, lung disease, coronary heart disease, heart failure, dementia, diabetes, stroke, aortic stenosis, and depressive symptoms

VO_2peak = peak oxygen consumption; PFS = Pittsburgh Fatigability Scale; PPFI = Pittsburgh Performance Fatigability Scale

Table S3. Associations of Sedentary Behavior with Perceived Physical Fatigability, Adjusted for Step Count/day: The Study of Muscle, Mobility and Aging (SOMMA, N=658)

	1 SD	β (SD)	95% CI
**Total Sedentary Time, min/day**			
Continuous (per SD) †	112	0.16 (0.36)	-0.54, 0.86
Q1 (lowest)[#]		Ref.	
Q2		0.86 (0.88)	-0.87, 2.59
Q3		-0.15 (0.91)	-1.93, 1.64
Q4 (highest)		1.15 (0.99)	-0.79, 3.09
**Mean Sedentary Bout Length, min/day**			
Continuous†	5.5	-0.49 (0.35)	-1.17, 0.19
Q1 (lowest)[#]		Ref.	
Q2		-0.27 (0.88)	-2.00, 1.46
Q3		0.05 (0.92)	-1.76, 1.86
Q4 (highest)		-0.79 (0.99)	-2.73, 1.16
**Sedentary Breaks, /day**			
Continuous†	13.2	0.43 (0.32)	-0.20, 1.05

†Separate models were generated using continuous and quartile sedentary behavior variables
*p<0.05
[#] Quartiles are compared to Q1 (Quartile 1). Quartile ranges for total sedentary time: Q1: 241.2467 to 547.6; Q2: >547.6 to 617.1081; Q3: >617.1081 to 691.5590; Q4: >691.5590 to 897.6678. Quartile ranges for mean sedentary bout length: Q1: 4.2183 to 11.2369; Q2: >11.2369 to 14.1469; Q3: >14.1469 to 17.7291; Q4: >17.7291 to 51.7430.

Notes: Linear regression models were adjusted for age, sex, clinic site, height (m), weight (kg), multimorbidity, absolute peak oxygen consumption (mL/min), and step count/day; Perceived physical fatigability was measured using the Pittsburgh Fatigability Scale Physical score (0-50, higher=greater fatigability)

Table S4. Associations of Sedentary Behavior with Performance Fatigability, Adjusted for Step Count/day: The Study of Muscle, Mobility and Aging (SOMMA, N=645)

	1 SD	β (SD)	95% CI
**Total Sedentary Time, min/day**			
Continuous†	111.7	-0.44 (0.15)*	-0.74, -0.14
Q1 (lowest)#		Ref.	
Q2		-0.69 (0.38)	-1.43, 0.06
Q3		-1.38 (0.39)*	-2.16, -0.61
Q4 (highest)		-1.12 (0.42)*	-1.95, -0.29
**Mean Sedentary Bout Length, min/day**			
Continuous†	5.4	-0.18 (0.15)	-0.48, 0.11
Q1 (lowest)#		Ref.	
Q2		-0.76 (0.39)	-1.52, 0.01
Q3		-0.51 (0.40)	-1.29, 0.28
Q4 (highest)		-0.80 (0.43)	-1.64, 0.04
**Sedentary Breaks, /day**			
Continuous†	13.2	-0.07 (0.14)	-0.35, 0.20

†Separate models were run using continuous and quartile sedentary behavior variables
*p<0.05
Quartiles are compared to Q1 (Quartile 1). Quartile ranges for total sedentary time: Q1: 241.2467 to 547.6; Q2: >547.6 to 617.1081; Q3: >617.1081 to 691.5590; Q4: >691.5590 to 897.6678. Quartile ranges for mean sedentary bout length: Q1: 4.2183 to 11.2369; Q2: >11.2369 to 14.1469; Q3: >14.1469 to 17.7291; Q4: >17.7291 to 51.7430.

Notes: Tobit regression models were adjusted for age, sex, clinic site, height (m), weight (kg), multimorbidity index, absolute peak oxygen consumption (mL/min), and step count/day; Performance fatigability was measured using the Pittsburgh Performance Fatigability Index (PPFI, range 0 to 100%, higher=greater performance deterioration)
